# Spontaneous Coronary Artery Dissection Secondary to Rheumatological Diseases: A Comprehensive Review

**DOI:** 10.7759/cureus.5231

**Published:** 2019-07-24

**Authors:** Waqas Ullah, Zain Ali, Shristi Khanal, Mariya Khan, Ganesh Gajanan, Yasar Sattar, Maryam Mukhtar, Vincent FIgueredo

**Affiliations:** 1 Internal Medicine, Abington Hospital-Jefferson Health, Abington, USA; 2 Internal Medicine, Civil Hospital Karachi, Dow University of Health Sciences, Karachi, PAK; 3 Internal Medicine, Icahn School of Medicine at Mount Sinai, New York, USA; 4 Internal Medicine, Fauji Foundation Hospital, Rawalpindi, PAK; 5 Cardiology, Saint Mary Medical Center, Philadelphia, USA

**Keywords:** coronary stenting, spontaneous coronary artery dissection, systemic lupus erythematosus

## Abstract

Spontaneous coronary artery dissection (SCAD) is a life-threatening condition and multiple conditions have been associated with this entity. This study aims to further investigate and characterize the association of the underlying rheumatological disease with SCAD. A comprehensive literature search on four databases was performed using different Medical Subject Headings (MeSH) and all articles on SCAD in association with rheumatological diseases were identified. The analysis was performed using the Statistical Package for Social Sciences (SPSS), v22 (IBM SPSS Statistics, Armonk, NY). Ten articles of SCAD secondary to rheumatological reasons were identified. The majority of presentations were associated with systemic lupus erythematosus (SLE). Most patients presented with a non-ST-elevation myocardial infarction (NSTEMI) involving the left main coronary vessel. The majority of them were successfully managed with stenting. Mortality was less than 20% with prompt identification and management of the SCAD. SLE was the most commonly reported rheumatological condition associated with SCAD. Prompt diagnosis and management of SCAD in such patients can be life-saving.

## Introduction

In 1956, Watson first introduced arterial dissection as a condition resulting from blood penetration into the arterial wall, causing a separation between the vessel layers, with or without a tear of the tunica intima (inner vessel layer) [1]. Spontaneous coronary artery dissection (SCAD) is defined as a tear in the coronary arterial wall resulting in blood dissecting between layers in the absence of atherosclerosis, traumatic, or iatrogenic injury [2]. It was first described by Pretty in a 42-year-old female in 1931 during a postmortem examination [3]. There are many conditions associated with SCAD, most notably with pregnancy, but little is known about its association with rheumatological and systemic inflammatory conditions. This study sought to determine the association of SCAD with rheumatological and systemic inflammatory conditions.

## Materials and methods

A literature search for relevant articles was performed on April 2, 2019, using MEDLINE (PubMed, Ovid), Embase, and Cochrane databases. There was no language or time restriction placed on the search. The search strategies included various combinations of Medical Subject Headings (MeSH) to generate two subsets of citations: one for SCAD, using MeSH terms like ‘spontaneous coronary artery dissection,’ ‘coronary artery dissection,’ ‘coronary artery rupture,’ ‘idiopathic coronary artery dissection,’ ‘SCAD,’ ‘spontaneous CAD,’ and the other for rheumatological conditions using MeSH, including ‘lupus,’ ‘SLE,‘ ‘systemic lupus erythematosus,’ ‘RA,’ ‘rheumatoid arthritis,’ ‘rheumatology,’ ‘rheumatological conditions,’ ‘connective tissue disorders,’ ‘systemic diseases,’ ‘polymyalgia rheumatica,’ ‘polymyositis,’ ‘psoriatic arthritis,’ ‘scleroderma,’ ‘Sjogren's syndrome,’ 'spondyloarthropathies,’ ‘tendinitis,’ ‘juvenile idiopathic arthritis,’ ‘infectious arthritis,’ ‘gout,’ ‘Crohn's disease,’ ‘bursitis,’ and ‘ankylosing spondylitis.’ The terms from the two subsets were combined in 1:1 combination, and finally, results from all the possible combinations were downloaded in full-text form. Based on our research question, we also manually searched the references in all known articles to identify studies that were missed by the initial search.

**Figure 1:**
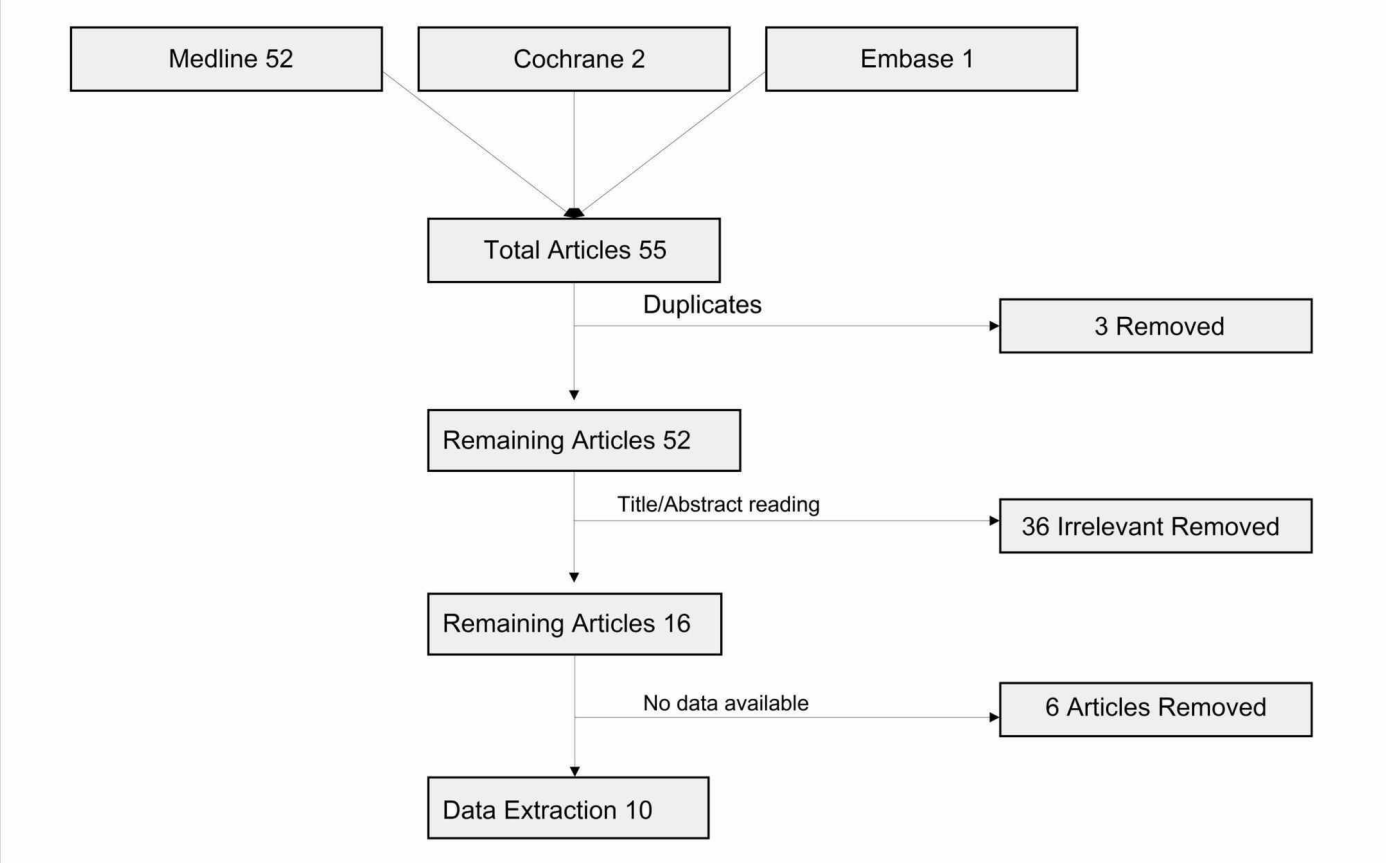
Preferred Reporting Items for Systematic Reviews and Meta-Analyses (PRISMA) flow sheet of the included studies

The titles and abstracts of the selected articles were reviewed independently by three authors and the articles which met the inclusion criteria (any rheumatological disease in association with SCAD) were reviewed by the fourth author (Figure 1). Data from the relevant articles were extracted into a Microsoft® Excel sheet (Microsoft Corp., Redmond, WA, USA) and were analyzed using the Statistical Package for Social Sciences (SPSS), v22 (IBM SPSS Statistics, Armonk, NY).

The combined systematic search strategy identified a total of 55 articles. After excluding duplicate and irrelevant articles items, 16 articles were deemed potentially eligible for data extraction. However, six articles did not contain sufficient data and were excluded. After final exclusions, 10 articles were identified for data analysis [4-13]. Figure 1 presents a Preferred Reporting Items for Systematic Reviews and Meta-Analyses (PRISMA) flowchart of the study selection process, along with reasons for study exclusion.

## Results

The mean age of included patients was 33 +/- 11 years (range: 17 - 49). Twenty percent were male, while 70% were female; the gender of one patient was not reported. Most patients presented with chest pain, and the signs and initial investigation were consistent with non-ST elevation myocardial infarction (NSTEMI) 40%, while one patient had ST-elevation myocardial infarction (STEMI) (Table 1). Interestingly, in one patient, the findings were incidental as the patient actually presented with diarrhea. There were no data available on the presentation of 30% of the patients.

**Table 1:** Frequency of Gender and Presentation of SCAD Patients NSTEMI: non-ST-elevation myocardial infarction; SCAD: spontaneous coronary artery dissection; STEMI: ST-elevation myocardial infarction

Gender	Frequency	Percentage
Male	2	20.0
Female	7	70.0
Total	10	100.0
Presentation	Frequency	Percentage
NSTEMI	4	40.0
STEMI	1	10.0
Incidental	1	10.0

**Figure 2:**
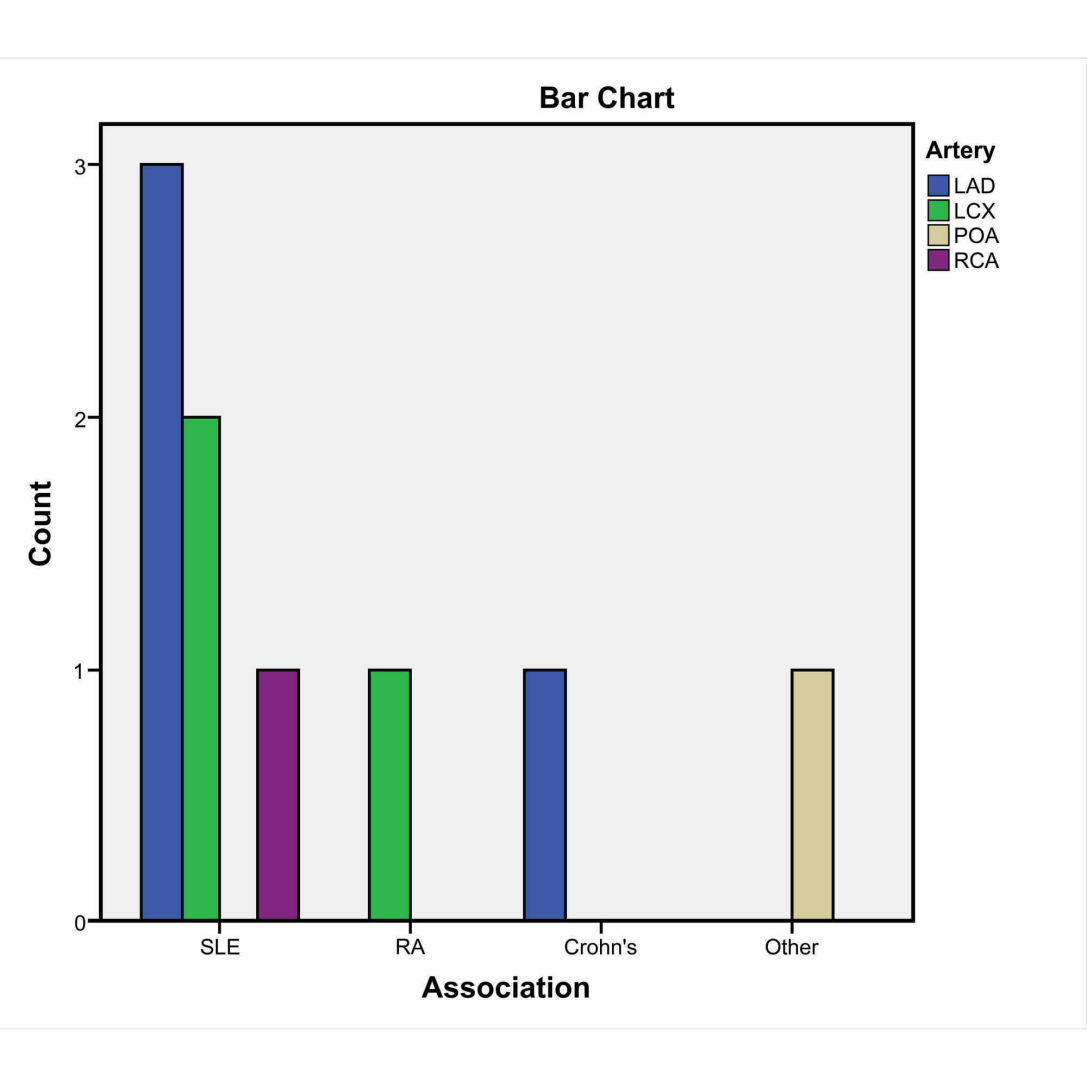
Type and frequency of different coronary artery involvements with the rheumatological disease

Amongst the site of artery involvement in our study population, 50% (n = 5) of patients had a coronary artery dissection of the left anterior descending artery (LAD), 30% (n = 3) had a dissection in the left circumflex artery (LCX), 10% (n = 1) had a dissection of the posterior descending artery (PDA), and the remaining 10% (n = 1) had a dissection of the right coronary artery (RCA) (Figure 2). In terms of associations, 60% of the patients with SCAD had an association with SLE. Rheumatoid arthritis (RA), Crohn's disease (CD), and other inflammatory diseases all had a 10% association with SCAD. In terms of the management of the dissection of coronary vessels in our study population, 60% of the coronary artery dissections were managed by coronary stents, 30% had no reporting of management data, and the remaining patients were managed by medications. In terms of associations, 60% of the patients with SCAD had an association with SLE. RA, CD, and other inflammatory diseases all had a 10% association with SCAD. In terms of the outcome of SCAD, 70% of the patients had a successful recovery, 10% of patients died, and the outcome was not reported in up to 20% of the cases. Artery involvement, associations, management, and outcomes are reported in Table 2.

**Table 2:** Association, Management, and Outcome of SCAD in Rheumatological Diseases LAD: left anterior descending; LCX: left circumflex; PDA: patent ductus arteriosus; RA: rheumatoid arthritis; RCA: right coronary artery; SCAD: spontaneous coronary artery dissection; SLE: systemic lupus erythematosus

Frequency	Percentage	Artery
5	50.0	LAD
3	30.0	LCX
1	10.0	PDA
1	10.0	RCA
Frequency	Percentage	Association
6	60.0	SLE
1	10.0	RA
1	10.0	Crohn’s
1	10.0	Polymyositis
Frequency	Percentage	Management
6	60.0	Stent
1	10.0	Conservative
Frequency	Percentage	Outcome
7	70.0	Survived
1	10.0	Died

**Figure 3:**
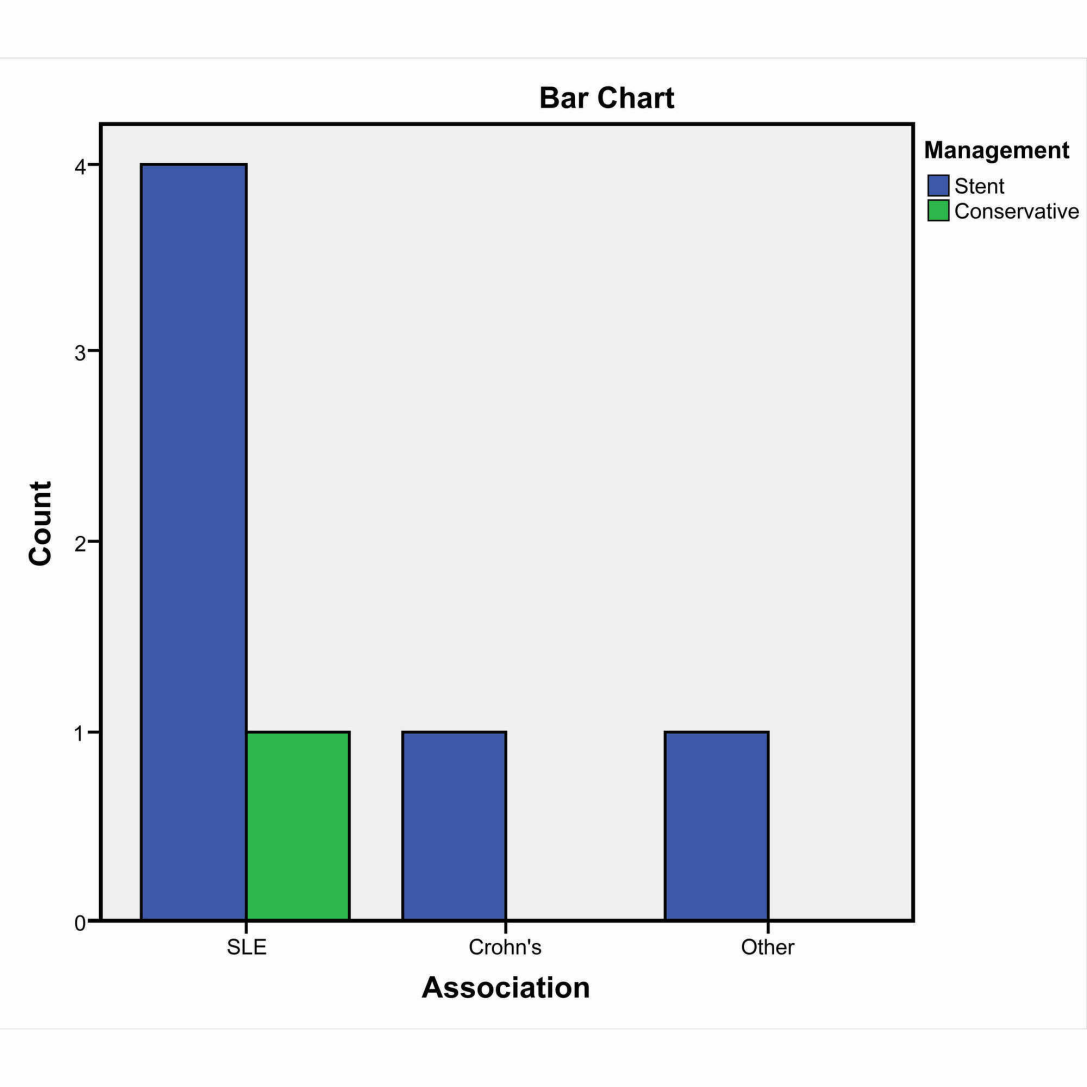
Management of spontaneous coronary artery dissection (SCAD) in association with rheumatological diseases SLE: systemic lupus erythematosus

We also sought to determine the association between the cause of SCAD and type of management and found that 80% of the patients had coronary stenting as the management of choice, while 20% were managed medically (Figure 3).

Pearson Chi-square analysis was utilized to determine the significance of this association. The likelihood ratio was 0.73, degree of freedom (df) was 2, and the p-value was 0.79 (non-significant). The likelihood ratio was used as the number of variables were more than 2 and the expected count in more than 20% of cells was less than 5. To determine the type of rheumatological disease (RD) predisposition to a specific coronary artery, we found that SLE was associated with LAD SCAD in 50% of cases and with LCX in 33% of cases. For all associations, the coronary artery involvement was not statistically significant, the likelihood ratio was 9.73, df was 9, and the p-value was 0.37 (Table 3).

**Table 3:** Likelihood Ratio of Management, Type of Artery, and Presentation of SCAD df: degree of freedom; SCAD: spontaneous coronary artery dissection

	Value	df	p-value
Presentation	10.41	4	.034
Management	.73	2	0.69
Artery	9.73	9	0.37

**Figure 4:**
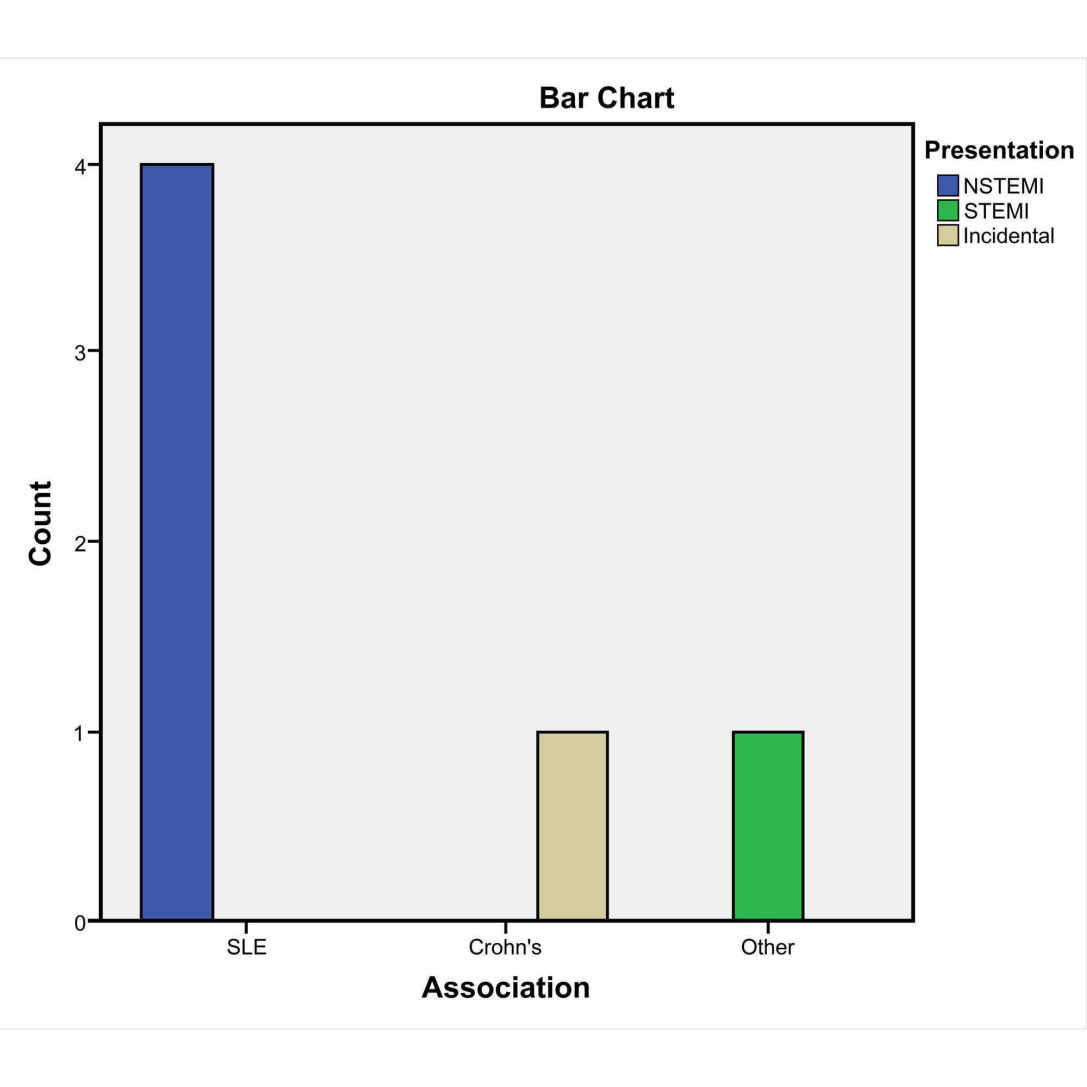
Association of SCAD with rheumatological disease and presentations NSTEMI: non-ST-elevation myocardial infarction; SCAD: spontaneous coronary artery dissection; SLE: systemic lupus erythematosus; STEMI: ST-elevation myocardial infarction

The most common presentation was NSTEMI. All patients with SLE had NSTEMI on presentation. Other presentations were not commonly reported. Interestingly, NSTEMI as a presentation of SCAD in RD was statistically significant with respect to other presentations, with a likelihood ratio of 10.4, df of 4, and a p-value of 0.03 (< 0.05). The estimated measured size for the strength of association was assessed with Cramér's V test and was found to be 1.0 (strongest association) (Table 3, Figure 4).

## Discussion

The true incidence of SCAD is unknown because of underdiagnosis of this rare condition. The estimated prevalence varies from 1% to 4% of all coronary angiograms [14]. Some other factors leading to underdiagnosis include inconclusive findings on the coronary angiography and the scarce literature on SCAD. SCAD is more commonly seen in a younger population, and women are more affected than men [15]. Vanzetto et al. reported that 80% of SCAD cases were female, with an average age of about 40 years [14]. It is the most common cause of myocardial infarctions in pregnant women [16]. This data was based on all cases of SCAD in the general population. Our study focused on SCAD in relation to rheumatological conditions. We found that, of the rheumatological diseases, SLE has the most common association with SCAD.

The mechanism of SCAD is thought to be an initial intimal tear, followed by intramural hematoma in tunica media, resulting in reduced coronary lumen size [17]. This compromises arterial supply to the myocardium, leading to myocardial infarction, arrhythmias, and sudden cardiac death [18]. Eighty percent of SCAD cases are related to other conditions, such as the peripartum state, connective tissue disease disorders, vasculitis, fibromuscular dysplasia, genetics, inflammatory conditions, autoimmune conditions, and hormonal therapy [19]. A national population-based survey reported that rheumatoid arthritis, systemic lupus erythematosus, and Crohn’s disease are the most common autoimmune conditions associated with SCAD [20]. However, the literature is scarce, not only due to limited reporting of these rare cases but also due to diagnostic challenges faced by physicians to identify these associations.

Recent clinical and experimental data suggest that the inflammation of the vessel wall, vasculitis, and increased risk of atherosclerosis might be the associated pathophysiology independent of cholesterol levels. Inflammatory markers, such as high-sensitivity C-reactive protein (hsCRP), have been found to be elevated in such cases [21]. Elevated hsCRP levels play a direct regulatory role in the process of atherosclerosis with the levels correlating with the severity of anatomical lesions and predicting adverse outcomes [22]. In our review of these cases of SCAD associated with rheumatological pathology, these inflammatory markers were not investigated. Only two cases of SLE reported serum antinuclear antibodies (ANA) and anti-double-stranded deoxyribonucleic acid antibodies (ds-DNA). There is now evidence that circulating hsCRP levels are closely linked to coronary artery events. Thus, it may be of value to investigate the extent of the effect of inflammatory mediators contributing to SCAD in rheumatological disorders.

SCAD should be suspected in patients presenting with acute coronary symptoms without the presence of traditional risk factors for coronary artery disease. The most common presentation in our study was NSTEMI. This presentation was so robust in our study that association by Cramér's V test was strongly positive. These findings highlight the fact that patients with atypical chest pain and subtle electrocardiogram (EKG) findings in the presence of rheumatological findings should be investigated for SCAD, especially in the female population. The characteristic findings and presentations of previously reported SCAD cases in association with the rheumatological diseases are provided in Table 4.

**Table 4:** Characteristics of Previously Reported Cases of SCAD Associated with Rheumatological Diseases AMI: acute myocardial infarction; ANA: antinuclear antibody; ds-DNA: double-stranded deoxyribonucleic acid antibodies; LAD: left anterior descending; LCX: left circumflex; N/A: not available; NSTEMI: non-ST-elevation myocardial infarction; PDA: patent ductus arteriosus; RA: rheumatoid arthritis; RCA: right coronary artery; SCAD: spontaneous coronary artery dissection; SLE: systemic lupus erythematosus; STEMI: ST-elevation myocardial infarction

Author/Ref	Age	Gender	Presentation	Artery involvement	Management	Rheumatological condition	ANA titer	DS DNA titer	Outcome
Reddy [4]	33	F	NSTEMI	LAD	Stent	SLE	N/A	N/A	survived
Rekik [5]	35	F	NSTEMI	LAD	stent	SLE	N/A	2:20	survived
Nisar [6]	31	M	NSTEMI	LCX	Medical management	SLE	N/A	112	
Sharma [7]	48	F	N/A	LAD	N/A	N/A	N/A	N/A	died
Besinger [8]	27	F	STEMI	PDA	Unsuccessful PCA	ill-defined inflammatory arthropathy	6:20	N/A	survived
Srinivas [9]	35	F	Bloody diarrhea	LAD	Stent	Crohn's disease	N/A	N/A	survived
Aldoboni [10]	39	F	N/A	LAD	Stent	SLE	N/A	N/A	survived
Kothari [11]	17	M	N/A	LCX		SLE	N/A	N/A	
Yoshikai [12]	49	F	AMI	RCA	stent	SLE	1:640	7.1	survived
Jajoria [13]	53	F	STEMI	LAD	stent	Polymyositis	N/A	N/A	survived

SCAD is diagnosed with coronary angiography. Findings on angiography in the majority of SCAD patients are different from the typical pattern. Usually, long and diffuse narrowing is seen on angiography due to an intramural hematoma, and these findings are usually inconclusive, leading to underdiagnosis of this condition [23]. In these scenarios, other modalities for a diagnosis, such as intracoronary imaging with optical coherence tomography (OCT), intravascular ultrasound (IVUS), or cardiac magnetic resonance imaging (MRI), should be considered [24]. Workup for other associated conditions, such as inflammatory conditions, connective tissue disease, hormone levels, autoimmune conditions, and fibromuscular dysplasia, should be considered once the patient is stabilized. Whole-body imaging is considered to rule out fibromuscular dysplasia, which is a frequent cause of SCAD [16].

Conservative management is usually chosen for clinically stable patients with a normal flow on angiography. This includes aspirin, beta blockers, clopidogrel, and statins. Spontaneous healing is often seen in patients managed conservatively [25]. If the patient has hemodynamic instability or continuous ischemic symptoms, percutaneous intervention (PCI) or coronary artery bypass graft (CABG) are considered. If the lumen is narrow and the flow is compromised, stent placement is tried, but it does have a risk of further dissection. Thrombolysis is associated with the expansion of dissection and is not considered as a treatment modality for these patients [25]. Our data showed that most patients were treated with PCI. Treatment of the underlying conditions, like SLE and RA, is also very important after the patient is stable. Molecular studies have shown that the anti-inflammatory effect of statins can be protective in coronary artery disease. It would be interesting to evaluate if there is a role for statins in preventing SCAD. Our analysis, however, did not find any mention of statins in SCAD management.

Even after management, patients experience recurrent SCAD, as well as other cardiovascular diseases. Patients managed conservatively have favorable hospital outcomes, whereas patients treated with revascularization have higher rates of treatment failure [26]. In a study by Saw et al., treatment failure was seen in 4.5% of the patients managed conservatively, whereas a 36.4% treatment failure rate was seen in patients managed with PCI [19]. Some studies have shown that 14% of patients require urgent in-hospital revascularization, usually because of the extension of dissection. During the follow-up at two to three years, major adverse cardiac events were reported in 10% to 30% of cases, mostly caused by recurrent MI and SCAD. Long-term follow-up showed major adverse cardiac events in almost 50% of patients [6]. Our data interestingly did not show any significant difference in the two treatment modalities. Ironically, most of the patients were treated with PCI and coronary stenting and had a favorable outcome.

Close follow-up should be done for the long-term as the recurrence of SCAD and other cardiovascular disease is high. Repeat coronary angiography should be used, however, only when the benefit outweighs the risk as it is associated with a higher risk of iatrogenic dissection [16].

Limitations

The data on rheumatological conditions associated SCAD is very scarce, and there are no randomized control trials or large scale studies on this topic. Thus, a meta-analysis could not be performed. The baseline characteristics and randomization of subjects in the included studies are unknown, and a temporal association and probable causation were reported in the included studies. This is why we can only assume that all patients with rheumatological conditions, and specifically SLE, can have a higher risk of SCAD. The role of inflammatory markers and its impact on coronary vessel wall is a hypothesis which needs a larger scale study.

## Conclusions

SLE was the most commonly reported rheumatological condition associated with SCAD. Physicians should consider this rare condition in patients presenting with NSTEMI and no obvious risk factors for coronary artery disease.
